# A single nucleotide polymorphism in *ADIPOQ* predicts biochemical recurrence after radical prostatectomy in localized prostate cancer

**DOI:** 10.18632/oncotarget.4980

**Published:** 2015-07-22

**Authors:** Chengyuan Gu, Yuanyuan Qu, Guiming Zhang, LiJiang Sun, Yao Zhu, Dingwei Ye

**Affiliations:** ^1^ Department of Urology, Fudan University Shanghai Cancer Center, Shanghai, China; ^2^ Department of Oncology, Shanghai Medical College, Fudan University, Shanghai, China; ^3^ Department of Urology, The Affiliated Hospital of Qingdao University, Qingdao, China

**Keywords:** biochemical recurrence, adiponectin, prostate cancer, radical prostatectomy, single-nucleotide polymorphism

## Abstract

Adiponectin has been implicated in prostate cancer (PCa) aggressiveness. However, the role of genetic variations in the adiponectin (*ADIPOQ*) gene in PCa progression remains unknown. To determine whether genetic variants in *ADIPOQ* are associated with the risk of biochemical recurrence (BCR) after radical prostatectomy (RP). We evaluated three common *ADIPOQ* polymorphisms in 728 men with clinically localized PCa who underwent RP. Multivariable Cox proportional hazards models and Kaplan–Meier analysis were used to assess their prognostic significance on BCR. The plasma adiponectin concentrations were measured by enzyme-linked immunosorbent assay. *ADIPOQ* rs182052 variant allele was associated with both increased risk of BCR [HR: 2.44; 95% confidence interval (CI): 1.57–3.79, *P* = 6×10^−5^] and decreased adiponectin level (β = −0.048, *P* = 0.004). Stratified analyses demonstrated that the association was more pronounced in men with higher visceral adipose tissue. Our data support that the *ADIPOQ* rs182052 SNP may be a predictive biomarker for BCR after RP by a possible mechanism of altering the adiponectin level. If validated, genetic predictors of outcome may help individualizing treatment for PCa.

## INTRODUCTION

There is extensive evidence implicating obesity in prostate cancer (PCa) progression. It is increasingly clear that obesity is associated with a greater risk of higher grade and late-stage disease at the time of diagnosis [1]. Also, evidence of the association between abdominal obesity and biochemical recurrence (BCR) following radical prostatectomy (RP) has expanded significantly in recent years [2].

Considering that adipose tissue is a metabolically active endocrine organ, one of the major links between obesity and aggressive PCa is aberrant secretion and signaling of adipokines [3]. The properties of visceral adipose tissue (VAT), subcutaneous adipose tissue (SAT) and periprostatic adipose tissue (PPAT) can be distinguished depending on the distribution of adipose tissue [4, 5]. Adiponectin is the most abundant adipokine with lower levels seen in individuals with unfavorable metabolic conditions such as visceral obesity [6]. Specifically, adiponectin levels were found to be reduced significantly in metastatic PCa patients versus those with organ-confined disease [7], suggesting its involvement in PCa progression. It has been shown to inhibit PCa cell proliferation and invasion via restraining epithelial-to-mesenchymal transition process [8].

To our knowledge, three *ADIPOQ* single nucleotide polymorphisms (SNPs) (rs182052, rs266729 and rs3774262) were shown to be associated with both PCa susceptibility and adiponectin levels [9, 10]. They have been found to either have potentially functionality [11] or are associated with PCa clinicopathologic characteristics [10]. It is conceivable that these SNPs might influence PCa clinical outcome through modulation of adiponectin levels, which promotes a favorable microenvironment for PCa progression. In this regard, we sought to explore their prognostic significance on BCR among men with localized PCa who underwent RP.

## RESULTS

Patient characteristics are shown in Table 1. The median follow up times were 36.3 and 37.7 months, and there were 79 (25.3%) and 100 (23.5%) patients experienced BCR in Study 1 and Study 2, respectively. PSA levels, pathologic stage, lymph node involvement and Gleason score were significantly associated with BCR in both cohorts (*P* < 0.016).

**Table 1:** Clinicopathologic characteristics of the study populations

Variables	Qingdao, N = 312			Shanghai, N = 426		
	BCR	Non-BCR	*P*	BCR	Non-BCR	*P*
Age, years	67 (52-79)	68 (46-80)	0.991	68 (58-79)	68 (46-82)	0.951
BMI, kg/m^2^						
< 24	60 (77.9)	181 (77.0)	0.870	46 (46.0)	176 (54.0)	0.162
≥ 24	17 (22.1)	54 (23.0)		54 (54.0)	150 (46.0)	
PSA, ng/ml						
≤ 20	40 (51.9)	161 (68.5)	0.008	39 (39.0)	210 (64.4)	< 0.001
> 20	37 (48.1)	74 (31.5)		61 (61.0)	116 (35.6)	
Gleason score						
≤ 7(3+4)	20 (26.0)	107 (45.5)	0.002	27 (27.0)	173 (53.1)	< 0.001
≥ 7(4+3)	57 (74.0)	128 (54.5)		73 (73.0)	153 (46.9)	
Pathologic stage						
T1-2	24 (31.2)	138 (58.7)	< 0.001	49 (49.0)	233 (71.5)	< 0.001
≥ T3	53 (68.8)	97 (41.3)		51 (51.0)	93 (28.5)	
Lymph node involvement						
Negative	59 (76.6)	216 (91.9)	< 0.001	75 (75.0)	304 (93.3)	< 0.001
Positive	18 (23.4)	19 (8.1)		25 (25.0)	22 (6.7)	
Follow-up, months	36.3			37.7		
Adiponectin levels, μg/ml				7.0 (4.2-14.3)	7.1 (4.1-14.6)	0.225

**Figure 1:**
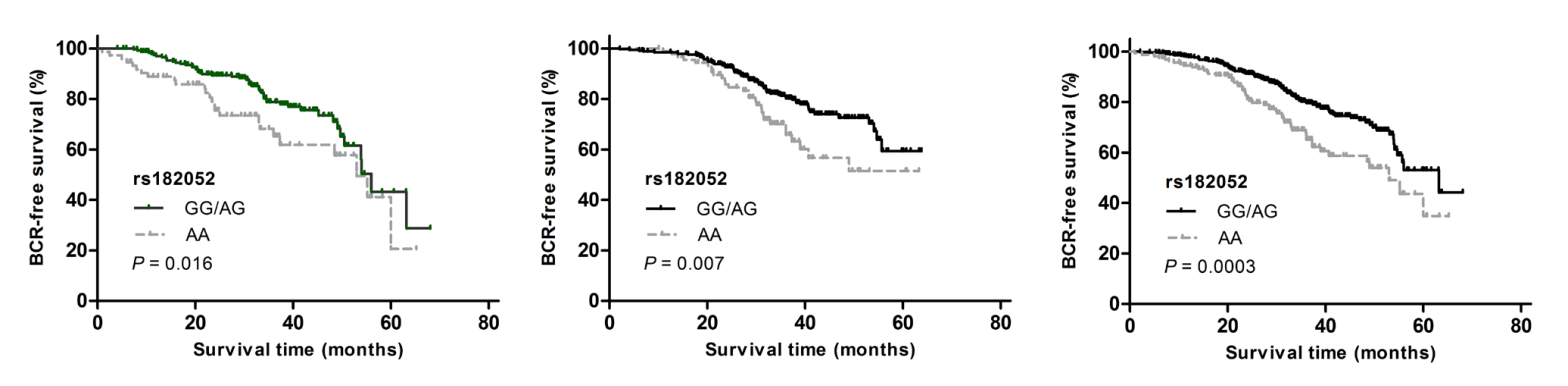
Kaplan-Meier survival curves for BCR-free survival according to *ADIPOQ* rs182052 by recessive model in (a) Study 1, (b) Study 2 and (c) combined analysis

In Study 1, we found a significant association of rs182052, but not rs266729 or rs3774262, with an increased risk of BCR (HR: 2.16, 95% CI: 1.07-4.38; Table 2). This association of rs182052 remained significant in Study 2 after adjusting for currently known clinical factors (HR: 2.39, 95% CI: 1.35-4.22). There was an elevation in risk of BCR with the number of variant A allele in both cohorts and combined analysis (log-rank *P =* 0.005, Figure 1A, 1B and 1C).

**Table 2:** Associations between *ADIPOQ* SNPs and BCR

**SNP**	Qingdao, N = 312				Shanghai, N = 426				Combined, N = 738			
	BCR, n (%)	Non-BCR, n (%)	HR (95% CI)^a^	*P*	BCR, n (%)	Non-BCR, n (%)	HR (95% CI)^a^	*P*	BCR, n (%)	Non-BCR, n (%)	HR (95% CI)^a^	*P*
rs182052												
GG	12 (15.6)	70 (29.8)	1.00		21 (21.0)	107 (32.8)	1.00		33 (18.6)	177 (31.6)	1.00	
AG	40 (51.9)	116 (49.4)	1.25 (0.64-2.41)	0.517	50 (50.0)	157 (48.2)	1.59 (0.95-2.65)	0.077	90 (50.8)	273 (48.7)	1.53 (1.02-2.28)	0.04
AA	25 (32.5)	49 (20.8)	2.16 (1.07-4.38)	0.032	29 (29.0)	62 (19.0)	**2.39 (1.35-4.22)**	**0.003**	54 (30.6)	111 (19.7)	**2.44 (1.57-3.79)**	**6**×**10^−5^**
AG/AA vs. GG			1.49 (0.79-2.80)	0.218			**1.81 (1.12-2.93)**	**0.016**			**1.75 (1.20-2.57)**	**0.004**
GG/AG vs. AA			**1.74 (1.08-2.80)**	**0.014**			**1.78 (1.14-2.77)**	**0.011**			**1.77 (1.28-2.45)**	**5**×**10^−4^**
rs266729												
CC	39 (50.6)	120 (51.1)	1.00		52 (52.0)	167 (51.2)	1.00		91 (51.4)	287 (51.2)	1.00	
CG	33 (42.9)	96 (40.9)	1.24 (0.78-1.98)	0.369	41 (41.0)	134 (41.1)	1.13 (0.75-1.71)	0.564	74 (41.8)	230 (41.0)	1.22 (0.90-1.66)	0.206
GG	5 (6.5)	19 (8.0)	1.48 (0.57-3.86)	0.423	7 (7.0)	25 (7.7)	1.09 (0.49-2.41)	0.831	12 (6.8)	44 (7.8)	1.16 (0.63-2.13)	0.635
CG/GG vs. CC			1.26 (0.80-1.99)	0.311			1.12 (0.76-1.67)	0.564			1.21 (0.90-1.63)	0.206
CC/CG vs. GG			1.17 (0.47-2.92)	0.743			1.04 (0.48-2.24)	0.929			1.03 (0.57-1.86)	0.920
rs3774262												
GG	35 (45.5)	104 (44.3)	1.00		41 (41.0)	143 (43.9)	1.00		76 (42.9)	247 (44.0)	1.00	
AG	37 (48.1)	114 (48.5)	1.13 (0.71-1.81)	0.609	50 (50.0)	156 (47.9)	1.44 (0.95-2.19)	0.085	87 (49.2)	270 (48.1)	1.34 (0.99-1.83)	0.062
AA	5 (6.4)	17 (7.2)	1.34 (0.51-3.55)	0.553	9 (9.0)	27 (8.2)	1.33 (0.64-2.77)	0.449	14 (7.9)	44 (7.9)	1.14 (0.64-2.02)	0.663
AG/AA vs. GG			1.15 (0.73-1.82)	0.546			1.49 (0.99-2.23)	0.051			1.31 (0.97-1.77)	0.077
GG/AG vs. AA			1.04 (0.42-2.60)	0.927			1.08 (0.54-2.17)	0.829			1.08 (0.54-2.17)	0.943

a Adjusted by age, PSA at diagnosis, Gleason score, pathologic stage and lymph node involvement.

To investigate the effect of SNPs on circulating adiponectin levels, we next evaluated differences in adiponectin levels by SNPs in Study 2. Consistent with previous report, plasma adiponectin levels were affected by rs182052. Individuals with rs182052 variant A allele had lower levels of plasma adiponectin (β = −0.048, *P* = 0.004). We did not observe significant differences in adiponectin levels by rs266279 and rs3774262 (β = 0.003, *P* = 0.867 and β = −0.033, *P* = 0.081, respectively).

In stratification analyses, we further assessed whether SNPs associations with BCR risk varied according to anthropometric measurements by the CT scan which were available for 208 patients (Table 3). The association between rs182052 and risk of BCR was more pronounced in men with higher VAT (HR: 2.53, 95% CI: 1.75-5.14; *P* = 0.01). The BCR and Non-BCR cases were similar with respect to the anthropometric measurements (Supplementary Table 1). There were no SNPs associated with these anthropometric measurements (Supplementary Table 2).

**Table 3:** Stratification analysis for associations between ***ADIPOQ*** SNPs and BCR by anthropometric measurements

Variables	rs182052 (BCR/Non-BCR)		HR (95% CI)^a^	*P*^a^	*P*^b^
	AA	AG/GG			
VAT					
< Median	3/23	14/64	1.15 (0.29-1.36)	0.837	**0.028**
> Median	15/11	21/57	**2.53 (1.75-5.14)**	**0.010**	
PPF					
< Median	10/12	23/59	1.79 (0.80-4.04)	0.158	0.913
> Median	7/18	13/66	1.58 (0.58-4.34)	0.372	
SAT					
< Median	9/13	21/60	1.24 (0.54-2.87)	0.608	0.053
> Median	8/17	15/65	2.04 (0.86-4.82)	0.106	
BMI					
< 24	10/31	36/145	1.01 (0.87-1.35)	0.281	0.622
≥ 24	11/31	43/119	1.09 (0.54-2.18)	0.808	

a Adjusted for age, BMI and smoking status.

b *P* value for homogeneity test.

## DISCUSSION

Our study among men with clinically localized PCa evaluated whether three established susceptibility loci in *ADIPOQ* influenced outcomes after RP. Presence of the minor allele in SNP rs182052 conferred a significant increased risk of BCR while controlling for known clinicopathologic risk factors, suggesting that this variant contribute independent data beyond currently used predictors. We found no associations between polymorphisms in the other two SNPs and BCR.

SNP rs182052 is located in the first intron of the *ADIPOQ* gene which contains a gene expression enhancer element [12]. *In silco* analysis showed rs182052 G>A changing confers a loss of a Sp1-binding site and gain of a CCAAT/enhancer-binding protein (C/EBP) β-binding site, which are both involved in adipocyte differentiation [13, 14]. This locus was identified by Dhillon et al. in a nested case-control study, whereby the A allele of rs182052 was associated with PCa predisposition [9]. The high-risk allele for Pca development aligned with worse prognosis, the plausible mechanisms of these associations may relate to circulating adiponectin levels.

In that respect, adiponectin is known to act as a potent inhibitor for PCa cells proliferation and invasion [15-17]. Adiponectin was shown to activate AMP-activated protein kinase (AMPK) in PCa cells, which in turn is involved in the anti-growth action via reduction in tumour protein translation [18]. Aside from AMPK, adiponectin is a potent inhibitor of PI3K/AKT /mTOR that is able to reduce cancer cell growth exerted by insulin and growth factor-induced signaling [19]. Circulating hormones and growth factors can also directly regulate tumor cell growth, or influence tumorigenesis through modifications of the stromal microenvironment composed of fibroblasts, immune-vascular and other cells [20]. For these reasons, a (genetically determined) modulation of adipoenctin in the tumor microenvironment could have a decisive role in determining prognosis for PCa.

Genetic studies have previously implicated the *ADIPOQ* locus for a role in influencing variation in adiponectin levels [21]. Very strong evidence for linkage of serum adiponectin levels to rs182052 has previously been found in two independent samples of Caucasian females from the Chingford Study and Twins UK cohort [22] and evidence for linkage has also been reported from at least four other European white populations [9, 23-25]. In Asian populations association tests also revealed significant association between the presence of the minor A allele and decrease in serum adiponectin [26, 27]. Consistent with these published data, we demonstrated that patients with rs182052 AA genetic background may maintain lower adiponectin levels than AG or GG carriers. Considering that rs182052 variant AA genotype was inversely associated with risk of BCR, these genotype-phenotype and genotype-risk associations were in the expected inverse directions to each other, suggesting a biological causal relationship.

Adipose tissue has an important role in more aggressive PCa since it display pivotal endocrine functions through the secretion of specific adipokines [3]. In the stratified analyses determined by body composition, we found indications of effect modification of rs182052 by high VAT. VAT and SAT are key contributors to abdominal obesity but differ in their structural composition, metabolic activity, and functional significance [4]. Individuals with increased VAT are at greater risk of developing metabolic disease, than are those with similar amounts of SAT [28]. The accumulation of VAT but not SAT mass is an independent predictor of reduced serum adiponectin levels [29]. The molecular mechanisms underlying the relationship may be that visceral adipocytes not only have lower levels of adiponectin expression but also reduced ability to functionally secrete this specific adipokine than subcutaneous adipocytes [30]. Thus, the adverse biologic functions of VAT may create a microenvironment favoring the development of more aggressive PCa. Thus, accounting for these risk factors and their potential interactions with genetic factors could result in larger discrimination in risk stratification. The absence of interaction with PPF suggests that other factors likely contribute to PCa recurrence, due in part to the high levels of IL-6 and VEGF in PPF [20].

In the current study, the rate of BCR was higher compared with previous American men studies [31]. On the other hand, BCR rates range from 10% to 35% in men undergoing RP worldwide [32] and our results were similar with studies in Asian countries [33-35]. One possible explanation for the rates of BCR differences relates to differential PSA screening situation. In China, where PSA screening is not as common as in the United States and Europe, many patients are diagnosed at more advanced stages. In the current study, 39.8% cases had stage 3-4 cancer. Advanced stages predict poorer prognosis after RP. In addition, cancer control rates following RP largely depend on the definition of BCR [32].

To the best of our knowledge, this is the first study to describe the association between genetic variations in the adiponectin gene with respect to clinical outcomes of PCa. Our data benefit from two independent patient populations from different regions of China with comprehensive adiposity phenotypes. In agreement with previous studies [36, 37], we found that PSA, Gleason score, lymph node involvement and disease stage were among the most important predictors of BCR. Thus, the results of this study are strengthened by these well-known prognostic factors of PCa recurrence. However, there are some limitations to consider in interpreting our study findings. First, the current findings are hypothesis generating, and the selected SNPs in our study did not fully cover the *ADIPOQ* gene. Although we chose to sequence three *ADIPOQ* SNPs based on previous study findings, some possible significant SNPs might be missed. A more comprehensive analysis of the *ADIPOQ* gene would be a future goal. Second, the relatively small sample size of the study might limit the power to reveal additional modest associations. Finally, our homogeneous Chinese Han population might make our findings less generalizable to other ethnic groups. Thus, our results warrant a larger-scale study with a longer follow-up period and in other ethnic populations for further validation.

We conclude that a polymorphism in the *ADIPOQ* gene is statistically significantly associated with clinical outcome in localized PCa patients received RP and this association may be modified by visceral obesity. Our results support that *ADIPOQ* genetic variations impact adiponectin exposure to potentially promote cancer growth and proliferation. Further functional studies are required to fully clarify the impact of variations in the *ADIPOQ* gene and PCa recurrence at the molecular level. Understanding the risk in individual patients will contribute to clinical prediction of prognosis, treatment options and approaches for secondary prevention.

## MATERIALS AND METHODS

### Study population

This study recruited 738 men with localized PCa who underwent RP from two centers in China. The first cohort (Study 1) consisted of 312 patients from the Affiliated Hospital of Qingdao University and Fudan University Shanghai Cancer Center between 2002 and 2005. The replication cohort (Study 2) is composed of 426 men recruited at Fudan University Shanghai Cancer Center between 2006 and 2009. All patients were followed up by serial PSA test every 3 months. Disease stage was determined according to criteria established by the American Joint Committee on Cancer (AJCC) tumor-node-metastasis (TNM) classification system [AJCC Staging Manual, sixth edition, 2002]. Histopathological grading of the RP specimens was performed according to the Gleason score system. The clinical information were abstracted from the archival medical records. RP patients who received adjuvant hormone therapy or radiotherapy were excluded. BCR was defined as 2 consecutive PSA measurements > 0.2 ng/mL at an interval of > 3 months, and the date of this event was set to the first of these two test occasions [38]. This study was approved by the Institutional Review Board of Fudan University Shanghai Cancer Center and the Affiliated Hospital of Qingdao University, and a written informed consent was obtained from each participant,

### Laboratory measurements

The *ADIPOQ* gene has two major haplotype blocks. We selected to genotype rs266729 (5′ flanking region), rs182052 (intron 1) to tag block 1 and rs3774262 (intron 2) to tag block 2. These PCa risk-associated SNPs were reported to be significantly associated with serum adiponectin simultaneously [9, 10]. Genotyping was done after extraction of DNA from whole blood using a standard QIAmp kit (QIAGEN Inc.) protocol using TaqMan real-time PCR method as described previously [10]. All SNPs had greater than 99% completion and the concordance was 100% for duplicated specimens. In 426 cases from FUSCC, plasma adiponectin was measured by an enzyme-linked immunosorbent assay (Abcam, USA) as reported previously [10].

### Anthropometric measurements

BMI ≥ 24 kg/m^2^ was defined as overweight according to the Chinese Working Group on Obesity guideline [39]. Abdominal VAT, SAT and PPAT were measured in preoperative computed tomography (CT) images in a subset of 208 patients using the average of 2 sections as standardized techniques. The quantities of abdominal VAT and SAT were measured at the level of the umbilicus (approximately the level of L4-L5) with patients in the supine position [40]. The measurement of PPF was performed on a transverse section at the level of caput femoris and greater trochanter of the femur [41]. The adipose area within the delineated contours of the CT was automatically calculated by ImageJ software based on predefined Hounsfield unit thresholds (−190 to −30).

### Statistical analysis

Continuous variables without normal distribution were compared by the Mann-Whitney U-test. Categorical variables were compared by the *x*^2^ test or Fisher's exact test. Adiponecin were ln-transformed to achieve a normal distribution. Hardy-Weinberg equilibrium (HWE) for evaluation of genotype frequencies was performed by the goodness-of fit χ2 test. All SNPs were in agreement with HWE (*P* = 0.63 for rs266729, 0.73 for rs182052 and 0.22 for rs3774262). The association between SNPs and PCa recurrence was assessed with hazard ratios (HR) and 95% CI estimated by Cox proportional hazards regression analysis under different genetic models. All analyses were adjusted for known prognostic factors including age, disease stage, Gleason score, lymph node involvement and PSA at diagnosis. The BCR-free survival interval was estimated using the Kaplan-Meier method, and the significance was determined using the log-rank test. Multiple linear regression analysis was used to examine the relationship between SNPs, adiponectin levels and anthropometric measurements. Anthropometric measurements were dichotomized based on their distribution in stratification analysis. All reported *P*-values are 2-sided. SAS version 9.1 (SAS institute Inc.) was used for all analyses.

## SUPPLEMENTARY MATERIAL TABLES


