# Supplementary material for: A single nucleotide polymorphism in ADIPOQ predicts biochemical recurrence after radical prostatectomy in localized prostate cancer
Source: Oncotarget. 2015 Jul 22;6(31):32205–11. doi: 10.18632/oncotarget.4980 (PMC4741670; doi:10.18632/oncotarget.4980)
Supplement: Supplementary file 1 [file oncotarget-06-32205-s001.pdf]

A single nucleotide polymorphism in *ADIPOQ* predicts biochemical recurrence after radical prostatectomy in localized prostate cancer

Supplementary Material

Supplementary Table 1. Anthropometric measurements of the study populations

Variables	BCR (N = 53)	Non-BCR (N = 155)	P^a
VAT, mm^2	17453 (4208-34855)	12010 (4107-34235)	0.001
SAT, mm^2	9564 (3580-31578)	11579 (2238-32955)	0.617
PPF, mm^2	2737 (1625-4799)	2987 (1798-5340)	0.031

[a]*P* value for Mann-Whitney's U-test.

Supplementary Table 2. Associations of SNPs with adiponectin level.

	SAT		PPF		VAT		BMI	
**SNP**	β	*P*	β	*P*	β	*P*	β	*P*
rs182052	-0.010	0.875	-0.018	0.089	-0.033	0.212	-0.027	0.598
rs266729	0.060	0.440	0.007	0.588	0.015	0.644	0.111	0.068
rs3774262	0.005	0.95	0.001	0.911	0.044	0.184	0.057	0.364

[a] Adjusting for age, BMI and statins use.
